# Selective hypermethylation is evident in small intestine samples from infants with necrotizing enterocolitis

**DOI:** 10.1186/s13148-022-01266-y

**Published:** 2022-04-11

**Authors:** Misty Good, Tianjiao Chu, Patricia Shaw, Lila S. Nolan, Joseph Wrobleski, Carlos Castro, Qingqing Gong, Olivia DeWitt, David N. Finegold, David Peters

**Affiliations:** 1 Division of Neonatal-Perinatal Medicine, Department of Pediatrics, University of North Carolina at Chapel Hill, 101 Manning Drive, Campus Box 7596, Chapel Hill, NC 27599 USA; 2 Department of Obstetrics, Gynecology and Reproductive Sciences, University of Pittsburgh, 204 Craft Avenue, Pittsburgh, PA 15213 USA; 3 Department of Pediatrics, Washington University School of Medicine, St. Louis, USA; 4 Department of Human Genetics, University of Pittsburgh, Pittsburgh, USA; 5 Department of Psychiatry, University of Pittsburgh, Pittsburgh, USA; 6 Magee-Womens Research Institute, Pittsburgh, USA

**Keywords:** DNA methylation, Epigenetics, Necrotizing enterocolitis, Intestine, Ileum, Neonatal

## Abstract

**Objective:**

Necrotizing enterocolitis (NEC) is the most common and lethal gastrointestinal disease affecting preterm infants. NEC develops suddenly and is characterized by gut barrier destruction, an inflammatory response, intestinal necrosis and multi-system organ failure. There is currently no method for early NEC detection, and the pathogenesis of NEC remains unclear.

**Design:**

To further understand the molecular mechanisms that support NEC, we used solution phase hybridization and next-generation DNA sequencing of bisulfite converted DNA to perform targeted genome-wide analysis of DNA methylation at high read depth.

**Results:**

We found that ileal samples from surgical NEC infants (*n* = 5) exist in a broadly hypermethylated state relative to their non-NEC counterparts (*n* = 9). These trends were not uniform, with hypermethylation being most consistently observed outside CpG islands and promoters. We further identified several biologically interesting gene promoters that displayed differential methylation in NEC and a number of biological pathways that appear dysregulated in NEC. We also found that DNA methylation patterns identified in ileal NEC tissue were correlated with those found and published previously in stool samples from NEC-affected infants.

**Conclusion:**

We confirmed that surgical NEC is associated with broad DNA hypermethylation in the ileum, and this may be detectable in stool samples of affected individuals. Thus, an epigenomic liquid biopsy of stool may have significant potential as a biomarker with respect to the diagnostic/predictive detection of NEC. Our findings, along with recent similar observations in colon, suggest that epigenomic dysregulation is a significant feature of surgical NEC. These findings motivate future studies which will involve the longitudinal screening of samples obtained prior to the onset of NEC. Our long-term goal is the development of novel screening, diagnostic and phenotyping methods for NEC.

**Supplementary Information:**

The online version contains supplementary material available at 10.1186/s13148-022-01266-y.

## Introduction

Necrotizing enterocolitis (NEC) remains one of the overall leading causes of death in premature infants in the neonatal intensive care unit (NICU) [1–3], and the precise etiology is not well understood. This devastating disease develops suddenly in 10% of premature infants, with half of the affected neonates requiring surgical excision of the necrotic intestine, and the associated mortality rate is as high as 50% [4]. Currently, there is no available method to predict the onset of NEC in at-risk infants. The treatment for NEC and the survival rate of infants afflicted by NEC have not changed over the past 40 years, with approximately $2–3 billion per year spent treating the disease [5–7].

A challenge in the management of NEC involves the absence of effective and predictive biomarkers, resulting in an inability to identify neonates at high risk of NEC development who may benefit from early preventative strategies. The importance of predicting NEC before the onset of disease is defined by three principles. First, by delineating the factors predictive of NEC, we will improve our understanding of the mechanisms and biologic pathways that lead to the exaggerated inflammatory response in NEC. Second, the use of predictors for NEC permits the identification of infants at the highest risk for whom an intervention may be tested and for whom intervention is most critically needed. The third motivation for the prediction of NEC is a corollary of the second; by identifying infants at low risk of NEC, unnecessary, costly, and sometimes hazardous interventions might be avoided.

A primary aim of this study was to determine the molecular phenotype of NEC through an epigenomic analysis of the ileum, with a specific focus on DNA methylation. Our interest in DNA methylation is motivated by its potential as a stool biomarker for early detection of NEC. Importantly, our previous studies have identified a significant degree of *global* hypermethylation in the NEC ileum via whole-genome bisulfite sequencing of laser captured enterocytes [8]. In this study, we aimed to expand these findings through *targeted* genome-wide analysis by solution phase hybridization to generate high read depth methylation sequencing data within well-characterized regions of the genome containing known genes and regulatory elements.

## Methods

### Study population and selection criteria

The intestinal samples in this study were collected in accordance with the University of Pittsburgh anatomical tissue procurement guidelines. Collection was approved by the Institutional Review Board (IRB) of the University of Pittsburgh (Protocols PRO09110437 or PRO14070508). Preterm infants were recruited under Protocol PRO09110437 at either Children’s Hospital of Pittsburgh (CHP) of University of Pittsburgh Medical Center (UPMC) or Magee-Womens Hospital Neonatal Intensive Care Units (NICUs), and consent was obtained from their parent or legal guardian. Resected intestinal tissue samples were obtained due to NEC or other non-inflammatory indications (such as re-anastomosis, spontaneous intestinal perforation or anorectal malformation). In some instances, deidentified intestinal samples were obtained with a waiver of consent and approval of University of Pittsburgh IRB (PRO14070508). For these cases, the clinical information obtained was limited to the location of the resected intestinal tissue sample and the surgical indication. Resections of intestinal tissue were snap-frozen and stored at -80 degrees Celsius until further analysis. Non-NEC tissue samples in this study are from patients after NEC had healed obtained during the surgical re-anastomosis. The use of healed NEC tissue obtained in this manner is standard in this field as infants can then serve as their own control. Complete information about neonatal sex for all surgically resected samples was unable to be obtained due to the deidentified nature of the way in which tissue was collected; therefore, we analyzed only autosomal genomic loci to minimize the impact of sex differences on our findings.

### DNA recovery from tissue sections

DNA recovery from ileal tissue sections was performed as previously described [9]. Snap-frozen specimens were mounted on appropriate embedding molds (Large, Thermo Scientific #2219; or Small, Sakura Tissue—Tek #4566) with clear OCT compound (Optimal Cutting Temperature Embedding Medium) (Fisher HealthCare #4585) and sectioned with a cryostat instrument (Leica CM 1850 UV, 7 microns). These sections were mounted on membrane slides (Leica PEN—Membrane Slide, 2.0 microns #11505158), stained with toluidine blue (Toluidine Blue 0.1% Aqueous, Newcomer Supply #14027), and air-dried (Sampla Dry Keeper, Samplatec. Corp). DNA was extracted from tissue sections using the Nucleospin Tissue XS Kit (Macherey–Nagel). Quantification of extracted DNA was performed with the KAPA hgDNA Quantification and QC Kit (Roche).

### Bisulfite sequencing

Bisulfite sequencing of solution phase captured DNA fragments was carried out as previously described [9]. DNA was sheared with Covaris to a size of ~ 175 bp. Libraries were prepared using the KAPA HyperPrep Kit (Roche). Libraries were bisulfite converted post-ligation using the EZ DNA Methylation-Direct Kit (Zymo) and amplified 13–15 cycles. Bisulfite-converted DNA libraries underwent targeted capture using the SeqCap Epi CpGiant Enrichment Kit (Roche, Pleasanton, CA) and amplified 10 cycles. Samples were sequenced on a HiSeq 2500 (Illumina) with 100 bp paired-end reads to a mean targeted read depth of ~ 24 ×. DNA sequence reads were quality trimmed and adaptor sequences were removed using Trim-Galore (https://www.bioinformatics.babraham.ac.uk/projects/trim_galore/). The reads were aligned to the human reference sequence (GRCh38/hg38) using Bismark Bisulfite Read Mapper [10] in paired-end, bowtie2 mode. Any unmapped reads were aligned in single-end, bowtie2 mode. Read duplicates were removed using Bismark. Methylation was called on paired-end and single-end files and then merged to determine the methylation status for each CpG site. DNA methylation signatures were determined using the beta-binomial test implemented in the R packages methylSig [11] and DSS [12].

## Results

Solution-phase hybridization was used to undertake targeted genome-wide bisulfite sequencing of DNA extracted from histological tissue sections of NEC ileum (*n* = 5) and non-NEC ileum (*n* = 9). We sequenced a total of 1,066,205,683 aligned read pairs across all samples. The targeted genomic regions collectively span approximately 80 Mb of the human genome and includes approximately 5.5 million CpG sites located within promoters, exons, introns, CpG islands (CGIs), CpG island shores and enhancers.

### Ileal NEC samples are broadly hypermethylated

In our targeted genomic analysis, we observed that ileal NEC samples had extensive DNA hypermethylation when compared to their non-NEC counterparts. This trend was evident in the broadest sense but also detectable when considering different genomic elements. The greatest effect was seen in regions without CGIs, whereas those regions containing CGIs were either less likely to be differentially methylated (CGIs in promoters) or displayed a bias toward hypomethylation in NEC (CGIs in introns/exons and CGIs in intergenic regions). These differences in the distribution of DNA methylation between NEC and non-NEC ileum samples were significant in all genomic contexts tested (Table 1).

**Table 1:** Proportion of CpG sites in different genomic elements that were hyper- or hypomethylated in NEC versus non-NEC control ileum

Region	Hypermethylated in non-NEC	Hypermethylated in NEC	Neither	Fraction hypermethylated in non-NEC	Fraction hypermethylated in NEC	*p* value
All Sites	25,616	71,961	1,990,463	0.013	0.036	NA
Prom No CGI	3776	15,571	315,307	0.012	0.049	0
Prom & CGI	585	782	240,903	0.002	0.003	0
Intron/Exon No CGI	10,259	32,288	701,345	0.015	0.046	0
Intergenic CGI	1391	1054	75,600	0.018	0.014	8.81 × 10^−274^
Intron/Exon & CGI	3198	2496	309,631	0.010	0.008	0
CGI Shores	1153	2874	78,909	0.015	0.036	6.79 × 10^−05^
Intergenic	5637	17,389	418,189	0.013	0.042	2.21 × 10^−93^

Only CpG sites with differing methylation levels between NEC and non-NEC by > 10% are included

**Fig. 1:**
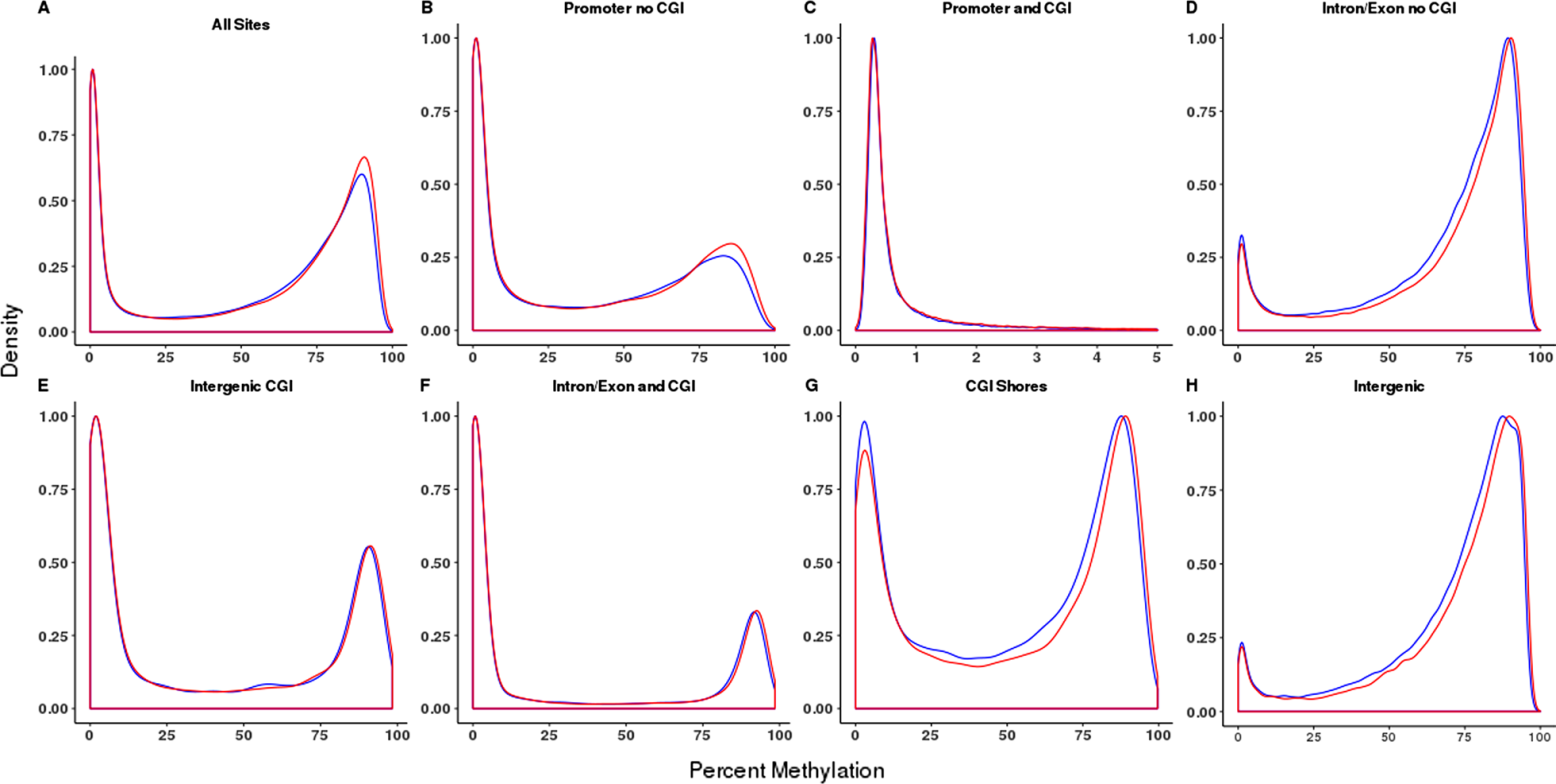
Distribution of methylation of non-NEC (blue) versus NEC (red) ileum by genomic element in **A** all CpG sites, **B** promoters without CGI, **C** promoter CGIs, **D** introns/exons without CGI, **E** intergenic CGI, **F** CGIs in introns/exons, **G** CGI shores, **H** intergenic regions without CGI. Note that only CpG sites read to a depth of 10 × or more were included in these analyses

**Fig. 2:**
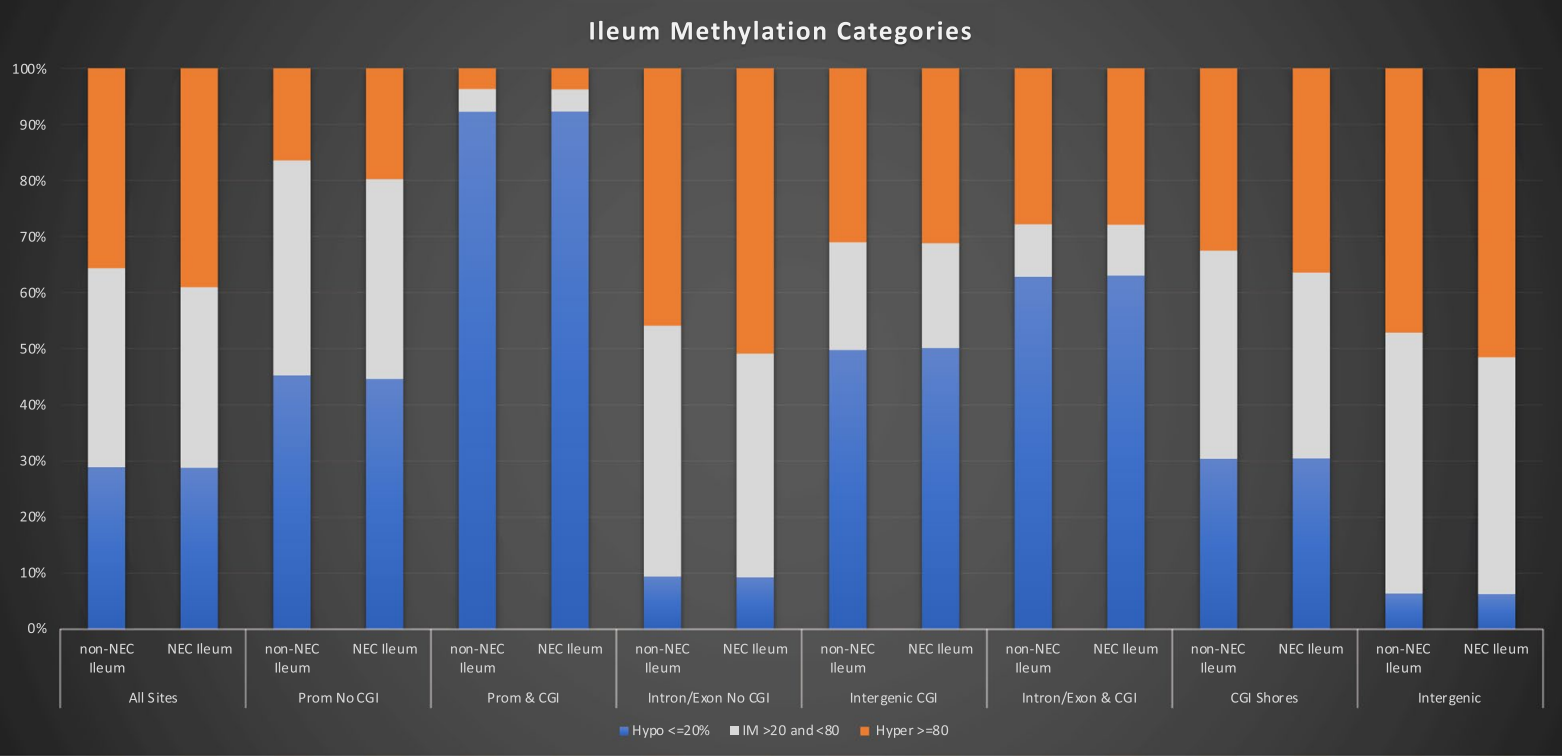
Comparison methylation category distribution (Hypo ≤ 20%, IM > 20% and < 80%, Hyper ≥ 80%) of non-NEC and NEC ileum by genomic element

A density plot analysis revealed a bimodal distribution of CpG methylation across all sites analyzed with peaks representing hypo- and hypermethylated states (Fig. 1A). These distributions varied when analyzing CpG sites in specific genomic regions. Specifically, introns, exons and intergenic regions that do not overlap with CGIs displayed a preponderance of hypermethylated sites (Fig. 1D and H). In contrast, CGIs in promoter regions contained very few hypermethylated CpG sites and, as expected, were largely hypomethylated (Fig. 1C). NEC-specific increases in CpG methylation were observed to varying degrees in all contexts, especially when all CpG sites were considered and in those genomic regions lacking CGIs (Fig. 1A, B, D, G, H and Table 2). DNA methylation levels were grouped into low (LM) (< 20%), intermediate (IM) (20–80%) or high methylation (HM) (> 80%) categories (Fig. 2) and distributions of these were altered between NEC and non-NEC ileum samples, reaching statistical significance for every genomic context tested (Table 3).

**Table 2:** Differences in distributions of DNA methylation between neonatal necrotizing enterocolitis and normal control samples within defined genomic region classes

Region	Diff.med (%)^a^	Diff.med (%)^b^	*p* value (W.holm)^c^
A: All Sites	0.60	1.31	0
B: Prom No CGI	0.46	1.58	0
C: Prom & CGI	0.01	0.08	1.19 × 10^−253^
D: Intron/Exon No CGI	1.40	1.74	0
E: Intergenic CGI	0.14	0.25	0
F: Intron/Exon & CGI	0.06	0.19	0
G: CGI Shores	0.79	1.27	0
H: Intergenic	1.37	1.66	0

CGI: CpG island

^a^Median difference between NEC ileum and non-NEC ileum

^b^Mean difference

^c^Adjusted for multiple comparisons using Holm’s method

**Table 3:** Distributions of low methylation (LM, ≤ 20%), intermediate methylation (IM, 20–80%) and high methylation (HM, ≥ 80%) in genetic elements (NEC vs. non-NEC)

Characteristics	Ileum	LM (%)	IM (%)	HM (%)	Chi-square *p* value non-NEC v. NEC
All sites	Non-NEC ileum	575,934 (28.93)	705,262 (35.43%)	709,267 (35.63%)	*p* < 2.2 × 10^−16^
	NEC ileum	573,632 (28.82)	640,256 (32.17%)	776,575 (39.01%)	
Prom No CGI	Non-NEC ileum	142,630 (45.24)	120,866 (38.33%)	51,811 (16.43%)	*p* < 2.2 × 10^−16^
	NEC ileum	140,603 (44.59)	112,387 (35.64%)	62,317 (19.76%)	
Prom & CGI	Non-NEC ileum	222,275 (92.27)	9796 (4.07%)	8832 (3.67%)	*p* = 0.03942
	NEC ileum	222,367 (92.31)	9508 (3.95%)	9028 (3.75%)	
Intron/Exon No CGI	Non-NEC ileum	66,057 (9.42)	313,258 (44.67)	322,030 (45.92)	*p* < 2.2 × 10^−16^
	NEC ileum	65,109 (9.28)	279,244 (39.82)	356,992 (50.90)	
Intergenic CGI	Non-NEC ileum	37,602 (49.74)	14,547 (19.24)	23,451 (31.02)	*p* = 0.02158
	NEC ileum	37,877 (50.10)	14,125 (18.68)	23,598 (31.21)	
Intron/Exon & CGI	Non-NEC ileum	194,501 (62.82)	28,989 (9.36)	86,141 (27.82)	*p* = 0.0002086
	NEC ileum	195,213 (63.05)	28,053 (9.06)	86,365 (27.89)	
CGI Shores	Non-NEC ileum	23,994 (30.41)	29,250 (37.07)	25,665 (32.52)	*p* < 2.2 × 10^−16^
	NEC ileum	24,064 (30.50)	26,084 (33.06)	28,761 (36.45)	
Intergenic	Non-NEC ileum	26,663 (6.38)	194,292 (46.46)	197,234 (47.16)	*p* < 2.2 × 10^−16^
	NEC ileum	26,253 (6.28)	176,432 (42.19)	215,504 (51.53)	

CGI: CpG island; LM: Low methylation; IM: Intermediate methylation; HM: High Methylation; NEC: Neonatal necrotizing enterocolitis

**Fig. 3:**
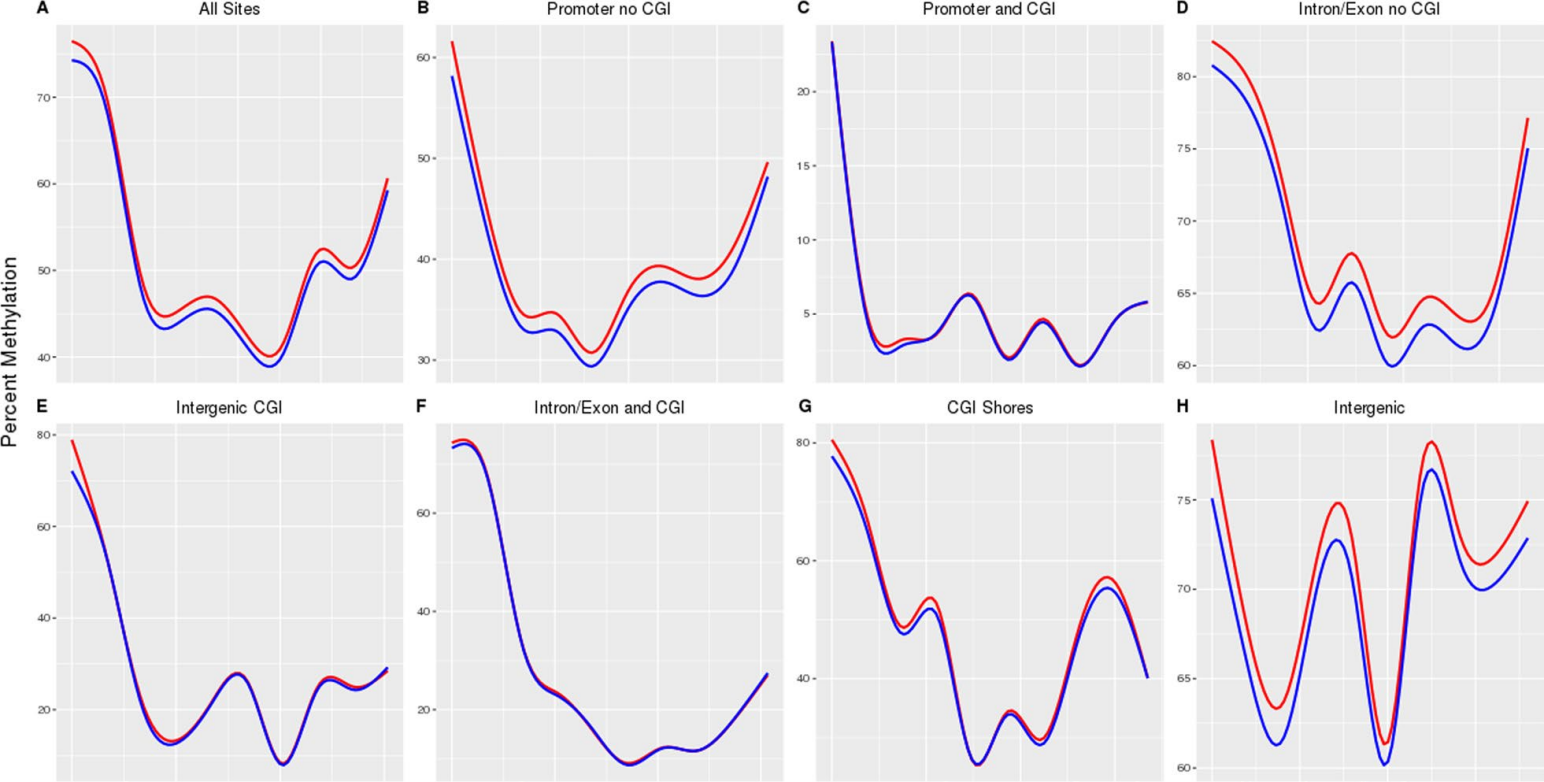
Non-NEC (blue) and NEC (red) methylation across Chromosome 1 broken down by genomic element

CpG methylation was further explored in a spatial context across each autosome, and we identified global hypermethylation of NEC ileum, relative to non-NEC controls, in regions that do not overlap with CGIs. As we previously observed, CpG methylation within CGIs was least affected by NEC regardless of CGI location (Fig. 3).

### Comparing NEC ileum methylation patterns with whole-genome bisulfite sequencing of laser captured ileal enterocytes

**Fig. 4:**
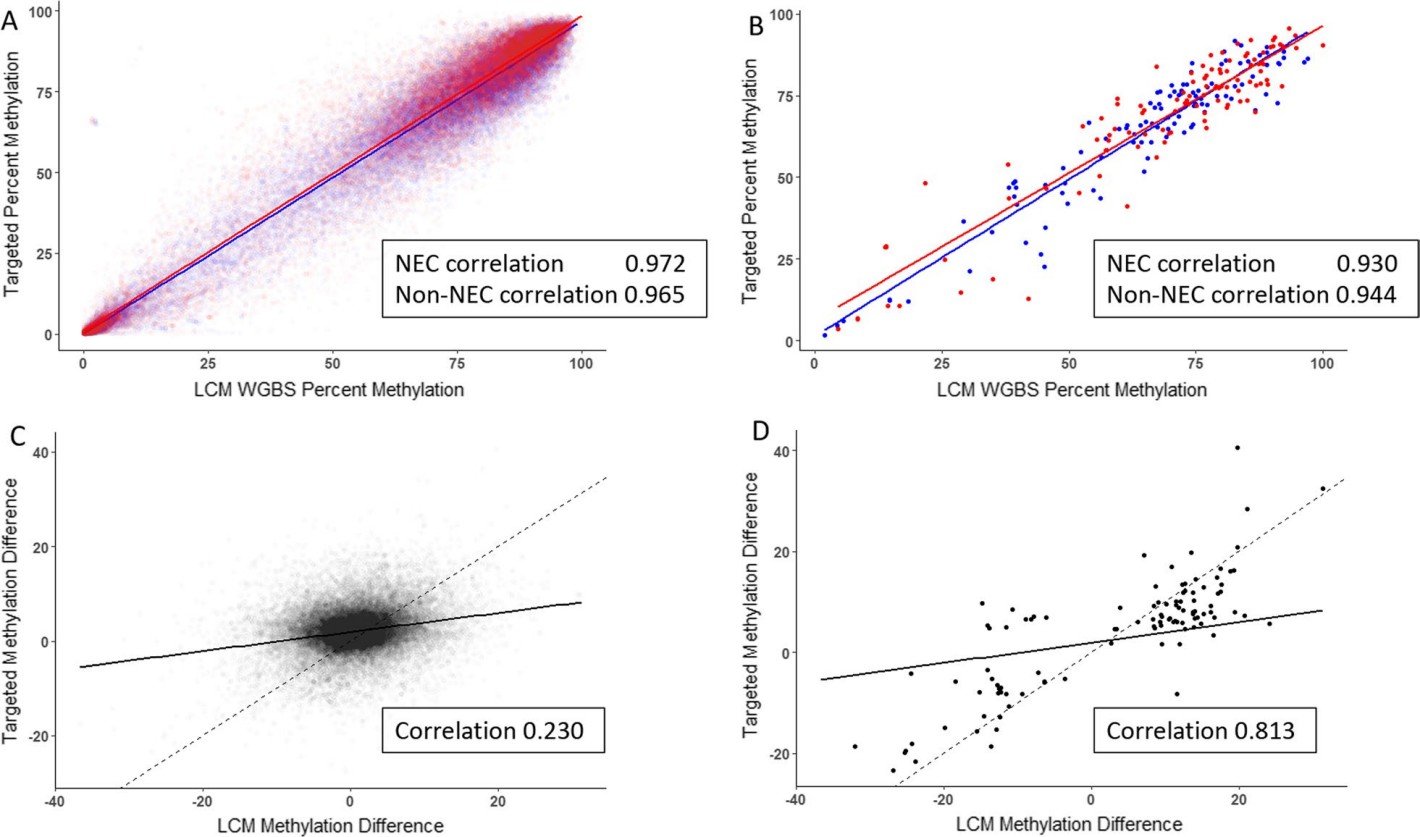
**A**, **B** Comparison of percent methylation between NEC (red) and non-NEC (blue) between ileum tissue and previously published data [8] from WGBS of LCM enterocytes. **A** All sites shared. **B** Shared sites with *p* < 0.05 in both methods. **C**, **D** Comparison of methylation difference (NEC minus non-NEC) between ileum tissue and WGBS of LCM enterocytes. **C** All sites shared. **D** Shared sites with *p* < 0.05 in both methods. In each case, the dashed line represents a correlation value of 1 between the two data sets

We next compared the results of the current study with previously published whole-genome bisulfite sequencing (WGBS) data, which were obtained by laser capture microdissection of ileal enterocytes from surgically resected NEC and non-NEC ileum [13]. A comparison between the current data and these previously published WGBS NEC ileum resulted in a correlation coefficient of 0.972 (*p* < 2.2 × 10^−16^) (Fig. 4). For non-NEC controls, the value was 0.965 (*p* < 2.2 × 10^−16^) (Fig. 4A). When we considered sites between NEC and non-NEC with a test p value of *p* < 0.05 in both data sets (laser capture/WGBS and current data), the correlation coefficients were 0.930 (*p* < 2.2 × 10^−16^) between NEC samples and 0.944 (*p* < 2.2 × 10^−16^) between non-NEC samples (Fig. 4B). We next compared the degree of CpG site-specific differential methylation between the two data sets and identified a correlation coefficient of 0.23 (*p* < 2.2 × 10^−16^) when all sites were considered (Fig. 4C) and 0.813 (*p* < 2.2 × 10^−16^) when only sites with a test *p* < 0.05 were considered (Fig. 4D). These results demonstrate that there is a considerable degree of correlation between the targeted genome-wide data presented herein and the data obtained following laser capture microdissection of enterocytes and WGBS.

### Gene-specific differential DNA methylation between NEC and non-NEC ileum

**Fig. 5:**
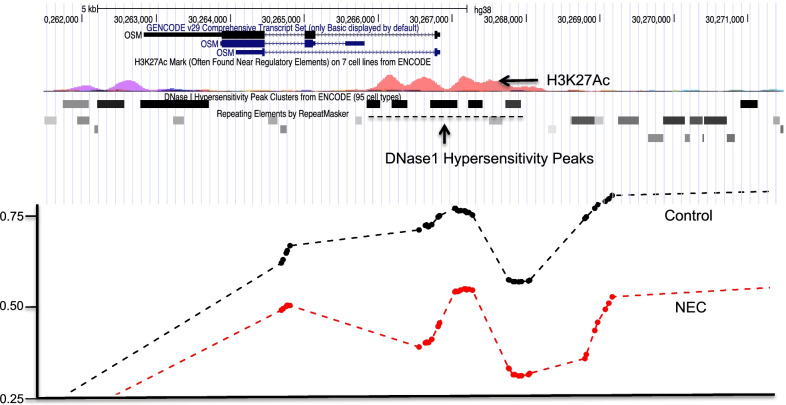
DNA methylation levels in the Oncostatin M (OSM) locus in ileum samples from NEC (red) and control (black) neonates. Differential methylation levels are shown across multiple CpG sites that cluster around regulatory elements in and around the flanking regions of the OSM gene locus

An analysis of NEC and non-NEC ileum identified numerous differentially methylated regions (DMRs). Specifically, we identified 1356 genomic loci contained within gene bodies (introns/exons) or promoter regions with a difference in average methylation rate of at least 0.1 between NEC and non-NEC ileum samples (Additional file 2: Table S1A). Of these, 974 were in promoter regions and could be broken down into those mapping to protein coding genes (*n* = 333, Additional file 2: Table S1B), long non-coding RNAs (lincRNAs) (*n* = 157, Additional file 2: Table S1C), micro-RNAs (miRNAs) (*n* = 75, Additional file 2: Table S1D) and antisense RNAs (*n* = 111, Additional file 2: Table S1E). We identified notable genomic loci, including Oncostatin M (OSM). As shown in Fig. 5, the OSM locus displays a reduction in CpG DNA methylation within its transcriptional regulatory region in NEC samples compared to non-NEC controls. OSM is a member of the interleukin-6 (IL-6) cytokine family and has been previously shown to drive intestinal inflammation in adults with inflammatory bowel disease [14], and a single nucleotide polymorphism in the OSM locus was associated with increased risk of IBD [15]. However, the role of OSM during NEC has not been previously described. OSM can induce signaling through the JAK-STAT pathway through the heterodimers gp130 and OSM receptor-β (OSMR) [14]. Additionally, we identified notable differentially methylated gene promoters, including GZMA (15% hypomethylated in NEC) [16], LTB4R (14.6% hypomethylated in NEC) [17], ISG20 (14.6% hypomethylated in NEC) [18], PLCB2 (12.7% hypomethylated in NEC) [19], VDR (12% hypomethylated in NEC) [20], TNIP1 [21] (19% hypermethylated in NEC) and GALNT6 [22] (22% hypermethylated in NEC). These are further discussed below.

We also identified 6793 differentially methylated single CpG sites (DMS) between NEC and non-NEC ileum samples using a filter of *p* ≤ 5 × 10^−3^. Of these, we found 943 in exons, 3405 in introns, 1996 in intergenic regions, 2116 in CGI shores and 22 in enhancers. We identified 484 located within CGIs and, of these, 76 of these were located within promoters, 143 in exons, 176 in introns and 156 in intergenic regions (Additional file 2: Table S2).

### Ingenuity pathways analysis of differentially methylated genes

Previous studies have demonstrated that DNA methylation signatures can reveal information about the molecular phenotypes of tissue or types of cells [23–25]. Therefore, we explored the functions of genes in which NEC-specific DMS were identified using Ingenuity Pathways Analysis (IPA) software. Specifically, we performed an IPA analysis on genes with promoters containing altered CpG methylation (*p* ≤ 0.05) in NEC versus non-NEC ileum samples. We identified the enrichment of genomic regions in numerous notable biological pathways under the control of a few known upstream regulators. For example, we found enrichment of genes in pathways including “RhoGDI Signaling” (*p* = 5.05 × 10^−6^), “FXR/RXR activation” (*p* = 5.35 × 10^−6^), “Hepatic Cholestasis” (*p* = 9.06 × 10^−5^), “Signaling by Rho Family GTPases” (*p* = 4.91 × 10^−4^) and “PXR/RXR Activation” (*p* = 5.29 × 10^−4^) (Additional file 1: Figures S1, Additional file 2: Table S3A). Predicted upstream regulators of these pathways include HNF1A (*p* = 6.55 × 10^−8^), TFRC (*p* = 2.79 × 10^−5^), STAT5A (*p* = 4.27 × 10^−5^), CBX5 (*p* = 8.18 × 10^−5^) and SMARCA4 (*p* = 1.29 × 10^−4^). Predicted downstream targets of these factors are shown in Additional file 2: Table S3B.

Given that DNA methylation in CGI shores has been shown to be associated with transcription [13], we next explored functional associations of genes containing differentially DMS in CGI shores whose DNA methylation levels were significantly altered between NEC and non-NEC ileum (*p* ≤ 0.05). We identified that these were enriched in several pathways including “molecular mechanisms of cancer” (*p* = 9.82 × 10^−9^), “GNRH signaling” (*p* = 6.51 × 10^−6^), “TGF-Signaling” (*p* = 6.56 × 10^−6^), “Thrombin Signaling” (*p* = 7.04 × 10^−6^) and “RhoGDI Signaling” (*p* = 1.72 × 10^−5^) (Additional file 1: Figure S2 and Additional file 2: Table S4A). Predicted upstream regulators included ERBB2 (*p* = 7.44 × 10^−7^), TGFB1 (*p* = 1.15 × 10^−5^), KDM1A (*p* = 2.89 × 10^−5^), ERG (*p* = 5.91 × 10^−5^) and SOX2 (*p* = 1.05 × 10^−4^). Predicted downstream targets of these factors are shown in Additional file 2: Table S4B.

We further performed IPA analysis of DMS within CGIs whose DNA methylation levels were significantly altered between NEC and non-NEC ileum (*p* ≤ 0.05). We found that CpG sites within CGIs whose methylation levels were significantly altered in NEC versus non-NEC ileum samples were in genes that were enriched in pathways including “Adipogenesis pathway” (*p* = 9.41 × 10^−6^), “CREB Signaling in Neurons” (*p* = 4.25 × 10^−5^) and “1D-myo-inositol Hexakisphosphate Biosynthesis II” (*p* = 4.41 × 10^−5^) (Additional file 1: Figure S3 and Additional file 2: Table S5A). Predicted upstream regulators of genes in these pathways included SOX2 (*p* = 1.41 × 10^−7^), POU5F1 (*p* = 9.75 × 10^−7^) and NANOG (*p* = 3.36 × 10^−5^), (Additional file 2: Table S5B).

### Comparison between ileal and colonic NEC-associated DNA methylation signatures

**Fig. 6:**
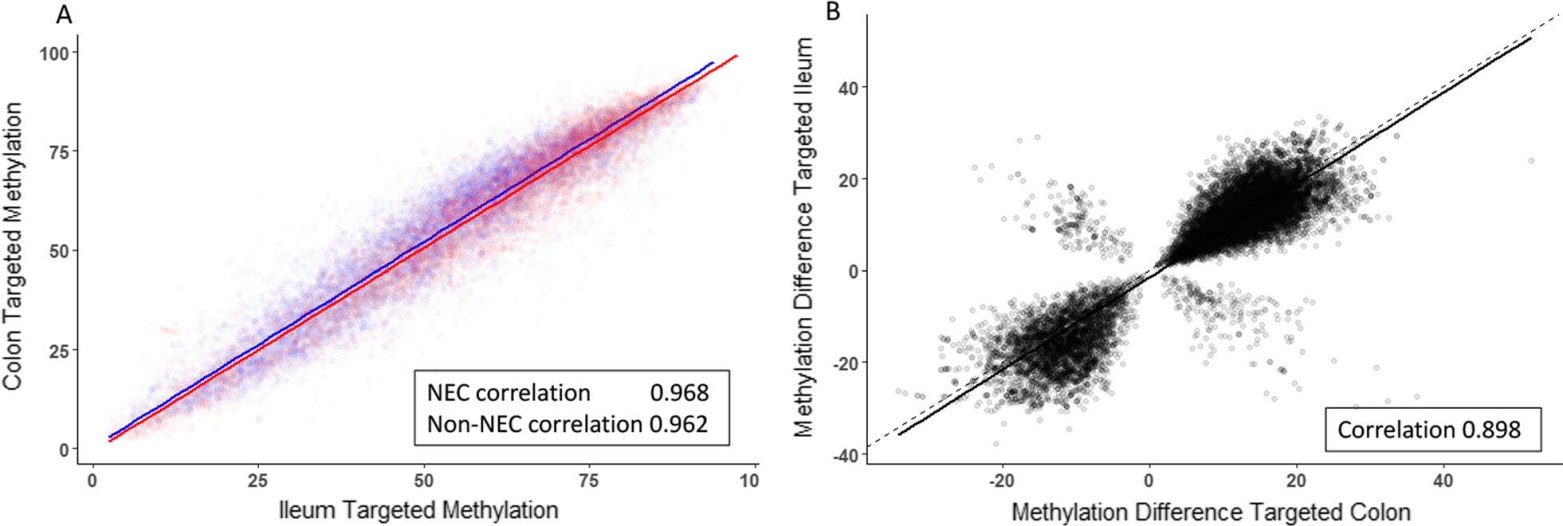
**A** Comparison of percent methylation between NEC (red) and non-NEC/control (blue) between ileum tissue and previously published colon tissue ^9^. Shared sites with *p* < 0.05 in both tissues. **B** Comparison of methylation difference (NEC minus non-NEC/control) between ileum tissue and colon. Shared sites with a *p* < 0.05 in both tissues. In each case, the dashed line represents a correlation value of 1 between the two data sets

We previously performed a comparative analysis of NEC and non-NEC colon tissue samples using comparable methods described herein for ileum. Comparisons between the current data and these previous data identified a high degree of correlation between non-NEC and NEC colon and ileum findings (Fig. 6). Among all sites targeted, the non-NEC and NEC correlation coefficient was 0.993 and 0.994, respectively, with a correlation of 0.564 for the difference (not shown). When only sites between NEC and non-NEC samples were considered with test *p* < 0.05, the correlation coefficients were 0.968 (*p* < 2.2 × 10^−16^) between NEC samples and 0.962 (*p* < 2.2 × 10^−16^) between non-NEC samples (Fig. 6A). A comparison of the degree of CpG site-specific differential methylation between the two data sets revealed a correlation coefficient of 0.898 (*p* < 2.2 × 10^−16^) when CpG sites with a test *p* < 0.05 were considered (Fig. 6B). These results demonstrate that there is a correlation between the targeted genome-wide data from the ileum presented herein and the data obtained from NEC and non-NEC colon tissue samples [9].

### A comparison of ileal and stool NEC-associated DNA methylation signatures

**Fig. 7:**
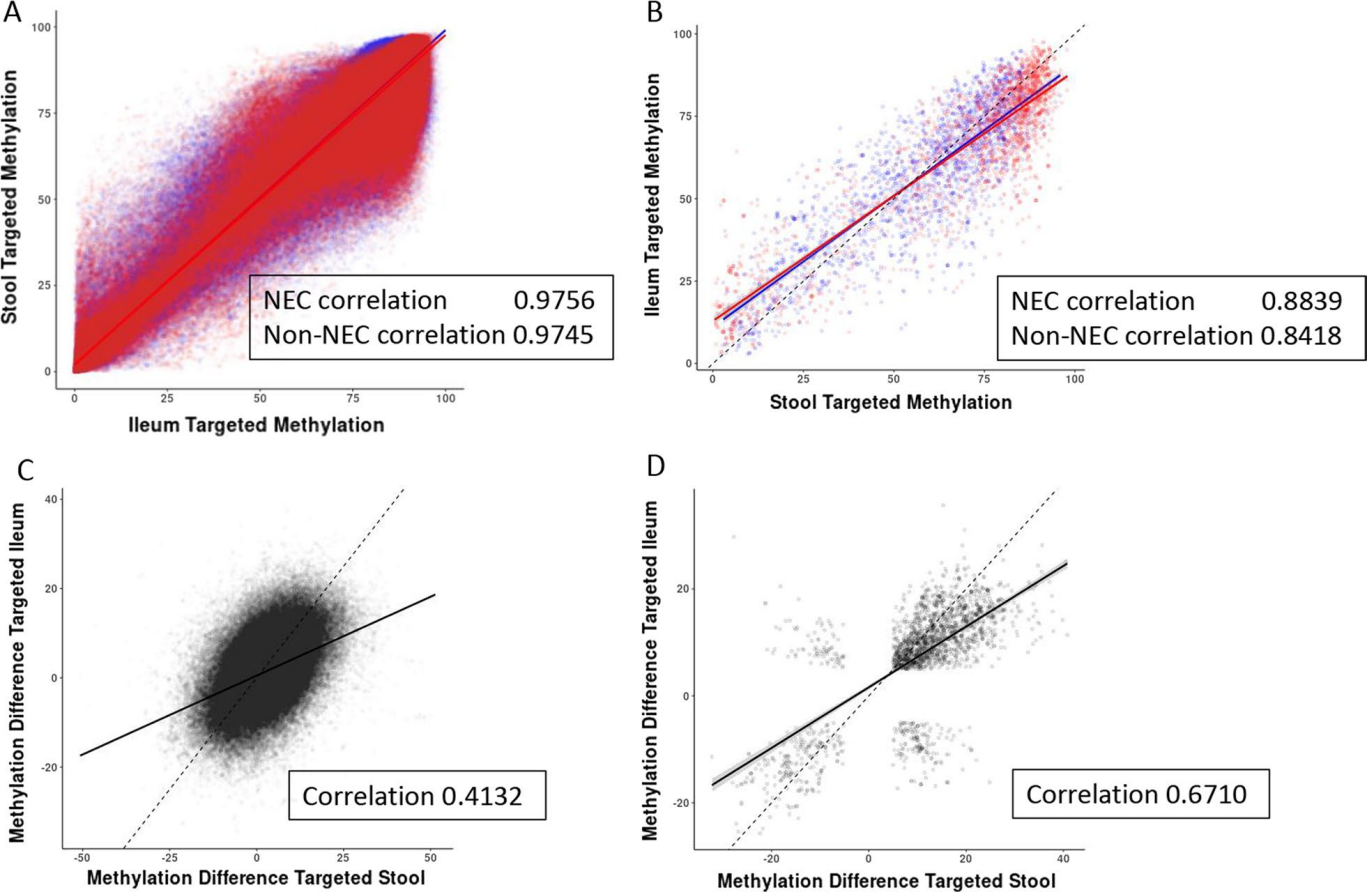
**A**, **B** Comparison of percent methylation between NEC (red) and non-NEC/control (blue) between ileum tissue and stool. **A** All sites shared. **B** Shared sites with a minimum absolute methylation difference of 5% or more and p < 0.05 in both methods. **C**, **D** Comparison of methylation difference (NEC minus non-NEC/control) between ileum tissue and stool. **C** All sites shared. **D** Shared sites with a minimum absolute methylation difference of 5% or more and *p* < 0.05 in both methods. In each case, the dashed line represents a correlation value of 1 between the two data sets

We next compared the DNA methylation signatures identified in NEC versus non-NEC ileum tissue with those obtained from stool samples from NEC and non-NEC infants. Stool data, which have been published previously [9], displayed clear hypermethylation in NEC samples compared to their non-NEC counterparts, and these trends are highly like those identified in ileal tissue from NEC and non-NEC infants. As shown in Fig. 7, we identified a significant correlation between NEC-associated DNA methylation signatures in tissue and stool.

## Discussion

The discovery of NEC biomarkers is an important step toward identifying means of early detection and improving overall management strategies. In this study, we present a comprehensive evaluation of DNA methylation in NEC ileum tissue samples compared to non-NEC controls. We observed that ileum samples from infants with surgical NEC exist in a broadly hypermethylated state, but these trends are not uniform across all types of genomic elements. The strongest trend was observed in genomic regions that do not contain CGIs, whereas regions containing CGIs showed more balanced DNA methylation between NEC and non-NEC ileum. The smallest difference was observed in CGIs located within promoters. These findings are consistent with our previous observations of DNA methylation in colon and ileum using whole-genome bisulfite sequencing [8]. However, because solution phase hybridization was used to target known genes and regulatory elements, the current study provides greater insight into the gene-specific changes in DNA methylation in NEC versus non-NEC tissue.

We also identified several notable genes and related biological pathways that are associated with NEC-specific differentially methylated loci. We further observed a significant correlation between NEC-specific DNA methylation differences identified in the ileum and those identified in both colon and stool. For example, we identified the promoter region of Granzyme A (GZMA), which was relatively hypomethylated in NEC versus non-NEC ileum. GZMA is known to be involved in intestinal epithelial cell detachment participating in the reduction of adhesion between epithelial cells and basement membranes, through its ability to cleave extracellular matrix components [16]. Similarly, the Leukotriene B4 receptor (LTB4R) promoter was also found to be hypomethylated in NEC samples. The protein encoded by this gene plays an important role in innate immunity, and the LTB4/LTB4R pathway is involved in the pathogenesis of the spectrum of human inflammatory diseases [17]. We noted that Interferon Stimulated Exonuclease Gene (ISG20) gene promoter was also hypomethylated in NEC samples. ISG20 has anti-viral properties and has been shown to be positively regulated by IL-22, an important cytokine involved in intestinal defense mechanisms [26] and recently shown to be a therapeutic strategy to attenuate inflammation in a pre-clinical model of NEC [27]. Inositol Polyphosphate-5-Phosphatase D (INPP5D, SHIP) was also hypomethylated in NEC. INPP5D deficiency is associated with ileitis and it is both involved in maintaining ileal microbial homeostasis and reduced levels have been reported in individuals with Crohn's disease [18]. Phospholipase C Beta 2 (PLCB2), which is involved in epithelial repair and integrity in the intestine [19], was also hypomethylated in its promoter region in NEC, as well as the vitamin D receptor (VDR), which protects against intestinal injury of NEC partly through suppressing the expression of the innate immune receptor, toll-like receptor 4 (TLR4) [20]. TNFAIP3 Interacting Protein 1 (TNIP1), the promoter of which was hypermethylated in NEC, helps to prevent intestinal inflammation by restricting intestinal epithelial cell death and preserving tissue integrity [21].

Notably, the trends identified in DNA methylation patterns of surgical NEC ileum samples were also detected in stool samples of affected infants. As observed in the ileal tissue, stool samples of NEC infants showed a broad degree of hypermethylation compared to their non-NEC counterparts. Furthermore, as in NEC ileal tissue, CpG sites in stool DNA were the least likely to be hypermethylated in NEC when they were in CpG islands in promoters. Also, as in NEC ileal tissue, the CpG sites in stool DNA that exhibited the greatest degree of hypermethylation in NEC were found to be in introns/exon sequences that do not contain CpG islands and intergenic regions.

The limitations of this case–control study include the inaccessible gender information for all surgically resected infants due to the nature of the IRB protocol and the relatively small sample size. Therefore, a further investigation of a larger cohort of both tissue and stool samples coupled with available clinical information is warranted. Furthermore, this was a cross-sectional study performed using NEC tissue and stool samples from infants with active NEC. Because there are no methods to predict the onset of NEC in premature infants, the rapid onset of NEC can result in tissue injury and necrosis. If the epigenomic changes that we identified in the ileal tissue and stool of infants with NEC are evident in early stages of NEC, this may provide an opportunity to develop non-invasive stool-based screening tools for its early detection. In particular, the early detection of NEC is key to the development of new therapeutics that can be implemented before the disease is clinically apparent. Future efforts will involve prospective studies of stool samples collected longitudinally so that we may explore whether DNA methylation changes appear prior to the onset of NEC. Future efforts will also explore whether hypermethylation, as we have observed in NEC, is a feature of other necrotized tissues.

## Conclusion

In conclusion, when comparing NEC tissues with non-NEC tissues, we observed broadly hypermethylated ileal NEC samples. We also found that broad DNA hypermethylation can be detected in stool samples from affected individuals. These findings, in addition to similar observations in the colon, suggest that epigenomic dysregulation is a significant feature of surgical NEC and may provide additional insights into NEC pathogenesis.

## Supplementary Information

**Additional file 1. Figure S1**: Enrichment of genes in (A) “RhoGDI Signaling” (p = 5.05 × 10^−6^), (B) “FXR/RXR activation” (p = 5.35 × 10^−6^) and (C) “Hepatic Cholestasis” (9.06 × 10^−5^). **Figure S2**: Enrichment of genes in (A) “molecular mechanisms of cancer” (p = 9.82 × 10^−9^), (B) “GNRH signaling” (p = 6.51 × 10^−6^) and (C) “TGF-Signaling” (p = 6.56 × 10^−6^). **Figure S3**: Enrichment of genes in (A) “Adipogenesis pathway” (p = 9.41 × 10^−6^), (B) “CREB Signaling in Neurons” (p = 4.25 × 10^−5^) and (C) “1D-myo-inositol Hexakisphosphate Biosynthesis II” (p = 4.41 × 10^−5^)

**Additional file 2**. Supplementary Data Tables.

## Data Availability

The datasets used and/or analyzed during the current study are available from the corresponding author on reasonable request.

## References

[1] Patel RM, Kandefer S, Walsh MC, Bell EF, Carlo WA, Laptook AR (2015). Causes and timing of death in extremely premature infants from 2000 through 2011. N Engl J Med.

[2] Anderson JG, Baer RJ, Partridge JC, Kuppermann M, Franck LS, Rand L (2016). Survival and major morbidity of extremely preterm infants: a population-based study. Pediatrics..

[3] Walsh MC, Bell EF, Kandefer S, Saha S, Carlo WA, D'Angio CT (2017). Neonatal outcomes of moderately preterm infants compared to extremely preterm infants. Pediatr Res.

[4] Hull MA, Fisher JG, Gutierrez IM, Jones BA, Kang KH, Kenny M (2014). Mortality and management of surgical necrotizing enterocolitis in very low birth weight neonates: a prospective cohort study. J Am Coll Surg.

[5] Bisquera JA, Cooper TR, Berseth CL (2002). Impact of necrotizing enterocolitis on length of stay and hospital charges in very low birth weight infants. Pediatrics.

[6] Stey A, Barnert ES, Tseng CH, Keeler E, Needleman J, Leng M (2015). Outcomes and costs of surgical treatments of necrotizing enterocolitis. Pediatrics.

[7] Neu J, Walker WA (2011). Necrotizing enterocolitis. N Engl J Med.

[8] Good M, Chu T, Shaw P, McClain L, Chamberlain A, Castro C (2020). Global hypermethylation of intestinal epithelial cells is a hallmark feature of neonatal surgical necrotizing enterocolitis. Clin Epigenet.

[9] Good M, Chu T, Shaw P, Nolan LS, McClain L, Chamberlain A (2021). Neonatal necrotizing enterocolitis-associated DNA methylation signatures in the colon are evident in stool samples of affected individuals. Epigenomics.

[10] Krueger F, Andrews SR (2011). Bismark: a flexible aligner and methylation caller for bisulfite-seq applications. Bioinformatics.

[11] Park Y, Figueroa ME, Rozek LS, Sartor MA (2014). Methylsig: a whole genome DNA methylation analysis pipeline. Bioinformatics.

[12] Park Y, Wu H (2016). Differential methylation analysis for bs-seq data under general experimental design. Bioinformatics.

[13] Good MCT, Shaw P, McClain L, Chamberlain A, Castro C, Rimer JM, Mihi B, Gong Q, Nolan LS, Cooksey K, Linneman L, Agrawal P, Finegold DN, Peters D (2020). Global hypermethylation of intestinal epithelial cells is a hallmark feature of neonatal surgical necrotizing enterocolitis. Clin Epigenet.

[14] West NR, Hegazy AN, Owens BMJ, Bullers SJ, Linggi B, Buonocore S (2017). Oncostatin m drives intestinal inflammation and predicts response to tumor necrosis factor-neutralizing therapy in patients with inflammatory bowel disease. Nat Med.

[15] Jostins L, Ripke S, Weersma RK, Duerr RH, McGovern DP, Hui KY (2012). Host-microbe interactions have shaped the genetic architecture of inflammatory bowel disease. Nature.

[16] Hirayasu H, Yoshikawa Y, Tsuzuki S, Fushiki T (2008). A lymphocyte serine protease granzyme a causes detachment of a small-intestinal epithelial cell line (iec-6). Biosci Biotechnol Biochem.

[17] Wilson GA, Butcher LM, Foster HR, Feber A, Roos C, Walter L (2014). Human-specific epigenetic variation in the immunological leukotriene b4 receptor (ltb4r/blt1) implicated in common inflammatory diseases. Genome Med.

[18] Dobranowski PA, Tang C, Sauve JP, Menzies SC, Sly LM (2019). Compositional changes to the ileal microbiome precede the onset of spontaneous ileitis in ship deficient mice. Gut Microbes.

[19] Lee SJ, Leoni G, Neumann PA, Chun J, Nusrat A, Yun CC (2013). Distinct phospholipase c-beta isozymes mediate lysophosphatidic acid receptor 1 effects on intestinal epithelial homeostasis and wound closure. Mol Cell Biol.

[20] Shi Y, Liu T, Zhao X, Yao L, Hou A, Fu J (2018). Vitamin d ameliorates neonatal necrotizing enterocolitis via suppressing tlr4 in a murine model. Pediatr Res.

[21] Kattah MG, Shao L, Rosli YY, Shimizu H, Whang MI, Advincula R (2018). A20 and abin-1 synergistically preserve intestinal epithelial cell survival. J Exp Med.

[22] Lavrsen K, Dabelsteen S, Vakhrushev SY, Levann AMR, Haue AD, Dylander A (2018). De novo expression of human polypeptide n-acetylgalactosaminyltransferase 6 (galnac-t6) in colon adenocarcinoma inhibits the differentiation of colonic epithelium. J Biol Chem.

[23] Chu T, Handley D, Bunce K, Surti U, Hogge WA, Peters DG (2011). Structural and regulatory characterization of the placental epigenome at its maternal interface. PLoS ONE.

[24] Chu T, Bunce K, Shaw P, Shridhar V, Althouse A, Hubel C (2014). Comprehensive analysis of preeclampsia-associated DNA methylation in the placenta. PLoS ONE.

[25] Reilly B, Tanaka TN, Diep D, Yeerna H, Tamayo P, Zhang K (2019). DNA methylation identifies genetically and prognostically distinct subtypes of myelodysplastic syndromes. Blood Adv.

[26] Klooster JPT, Bol-Schoenmakers M, van Summeren K, van Vliet ALW, de Haan CAM, van Kuppeveld FJM (2021). Enterocytes, fibroblasts and myeloid cells synergize in anti-bacterial and anti-viral pathways with il22 as the central cytokine. Commun Biol.

[27] Mihi B, Gong Q, Nolan LS, Gale SE, Goree M, Hu E (2021). Interleukin-22 signaling attenuates necrotizing enterocolitis by promoting epithelial cell regeneration. Cell Rep Med.

