# Supplementary figures and images for: Selective hypermethylation is evident in small intestine samples from infants with necrotizing enterocolitis
Source: Clin Epigenetics. 2022 Apr 11;14:49. doi: 10.1186/s13148-022-01266-y (PMC8996588; doi:10.1186/s13148-022-01266-y)

Figure S1A

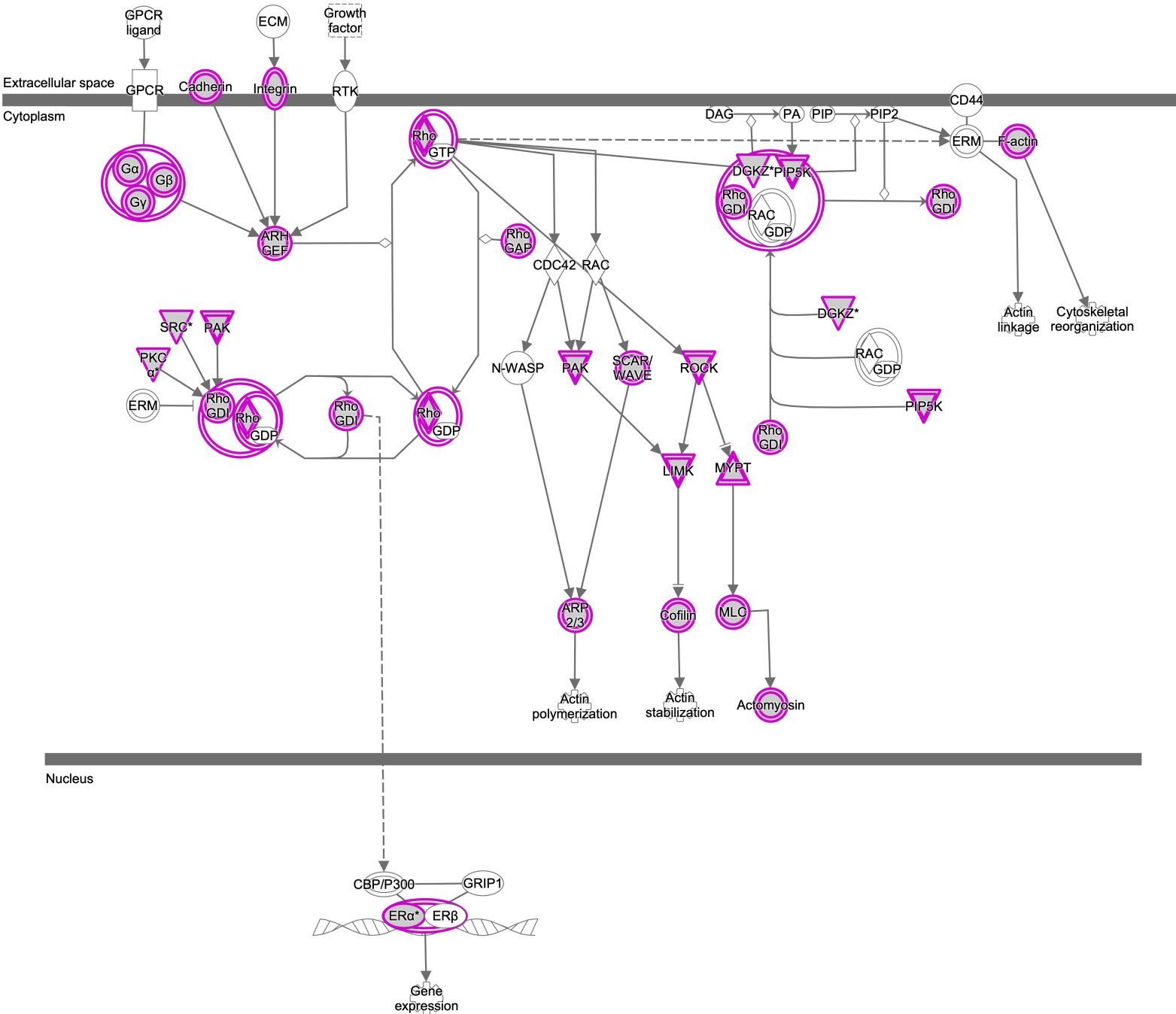

Figure S1B

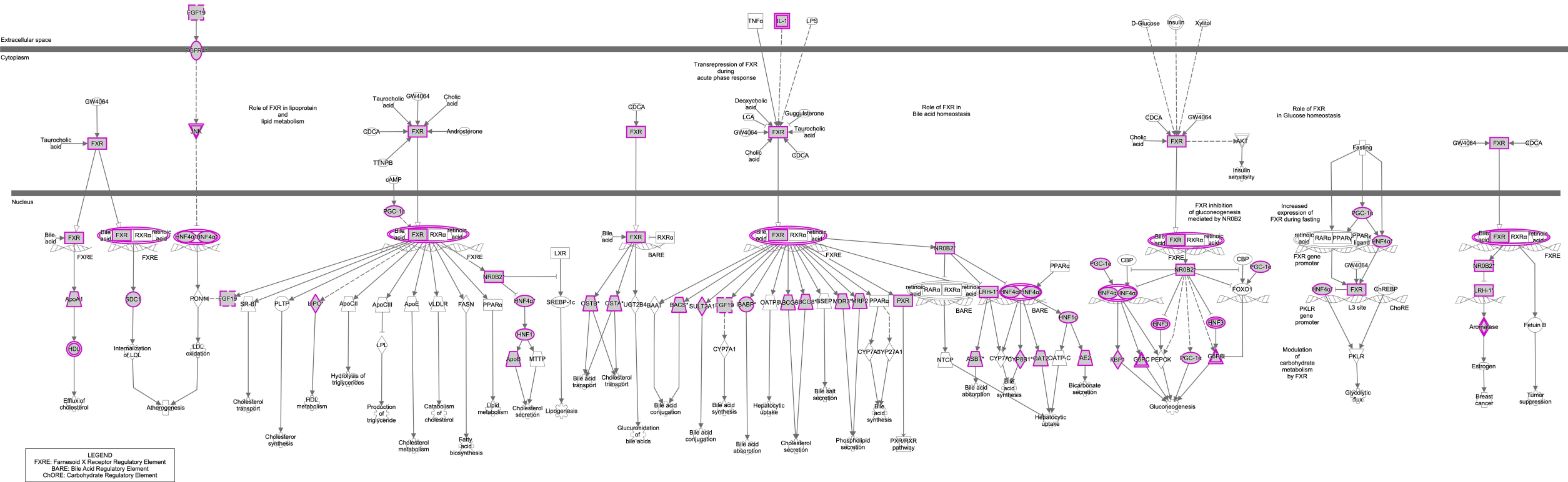

Figure S1C

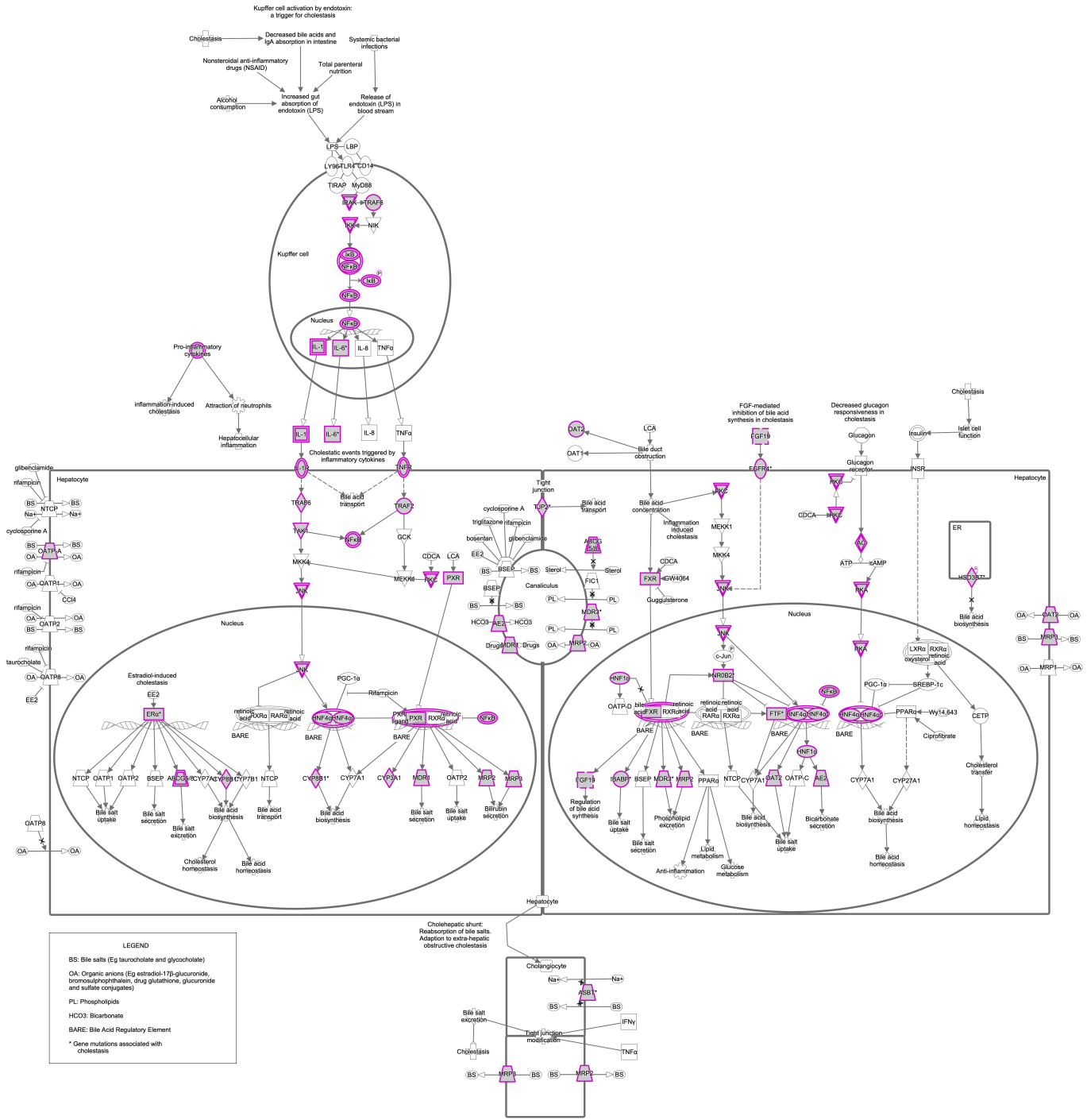

Figure S2A

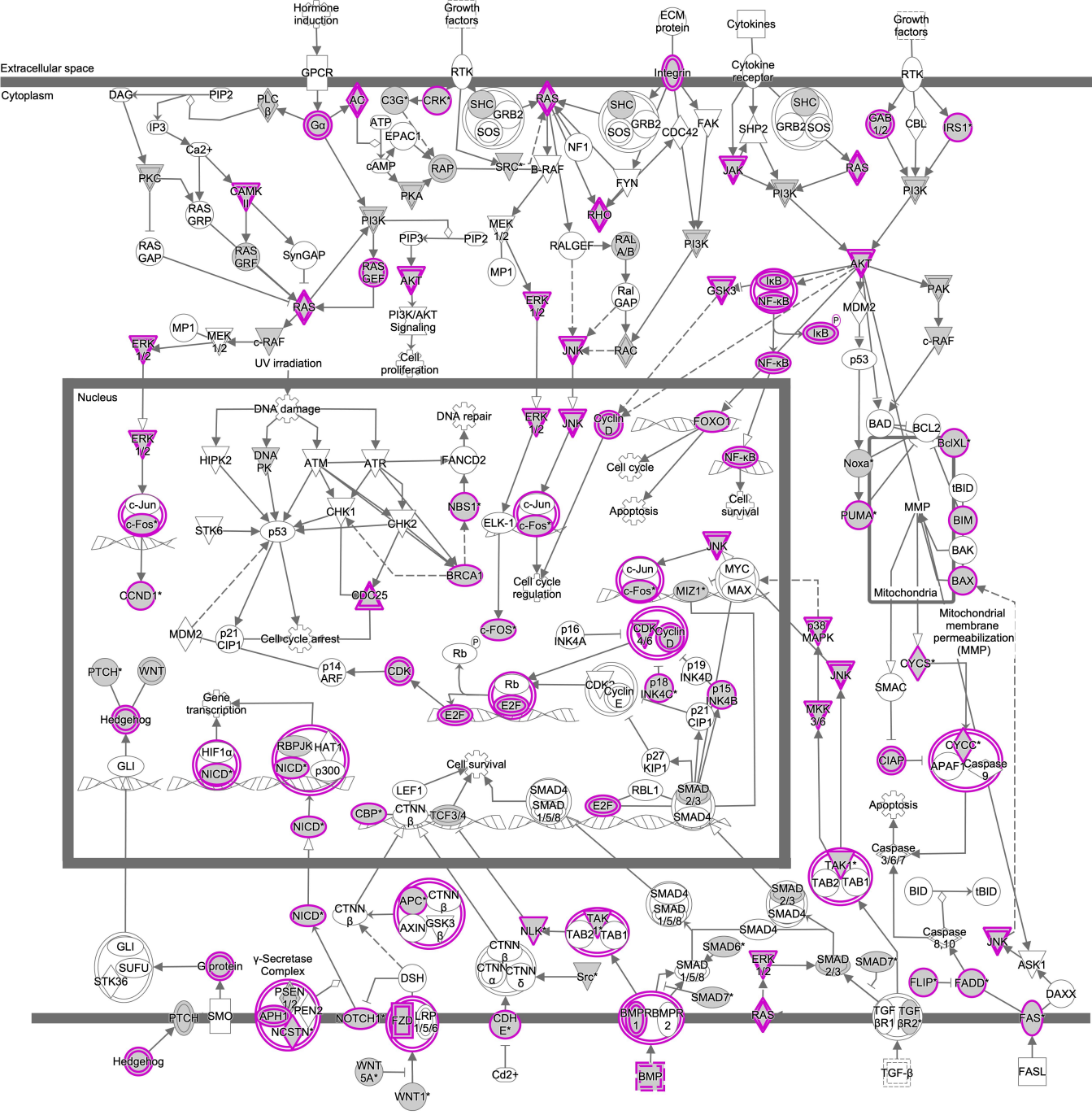

Figure S2B

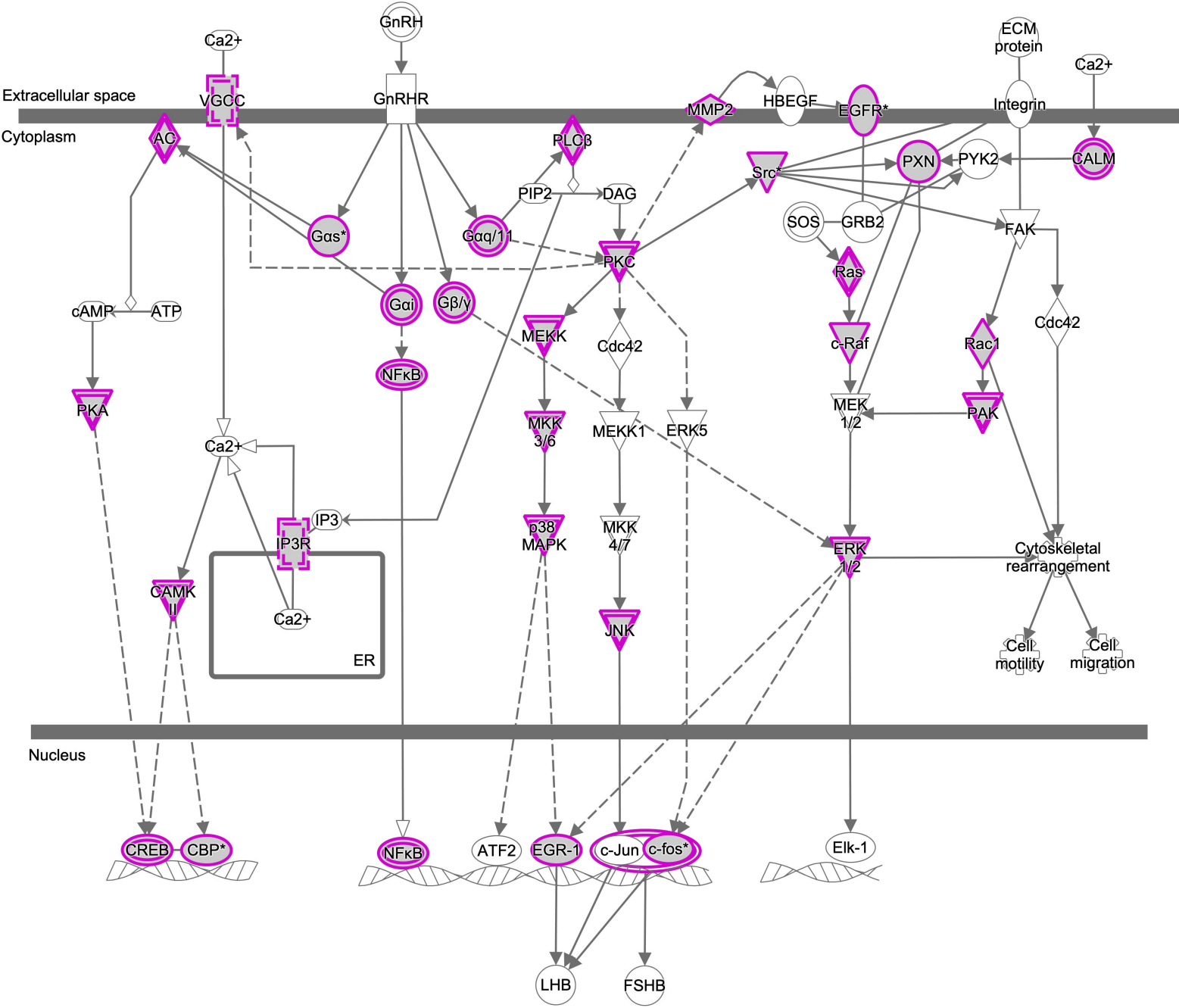

Figure S2C

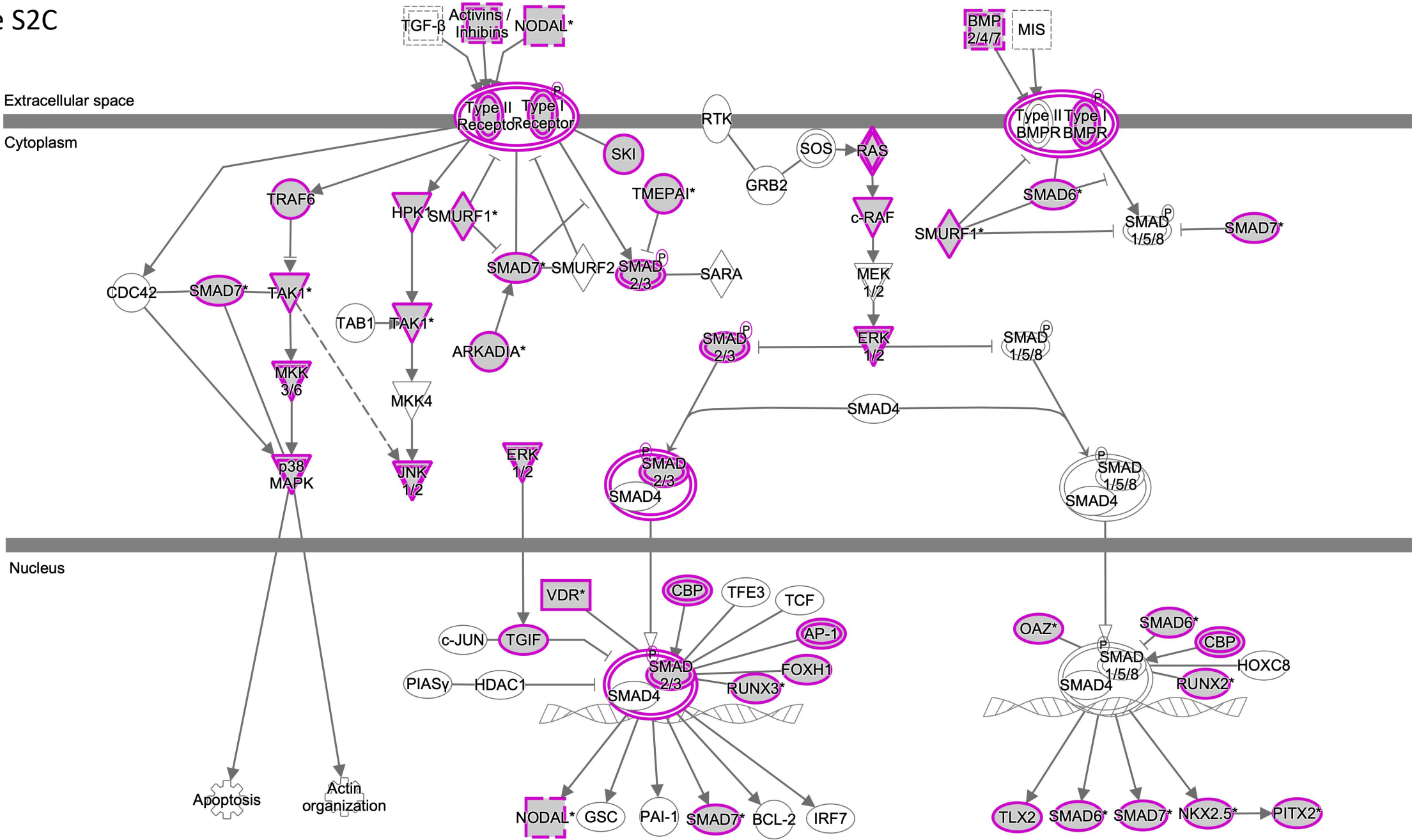

Figure S3A

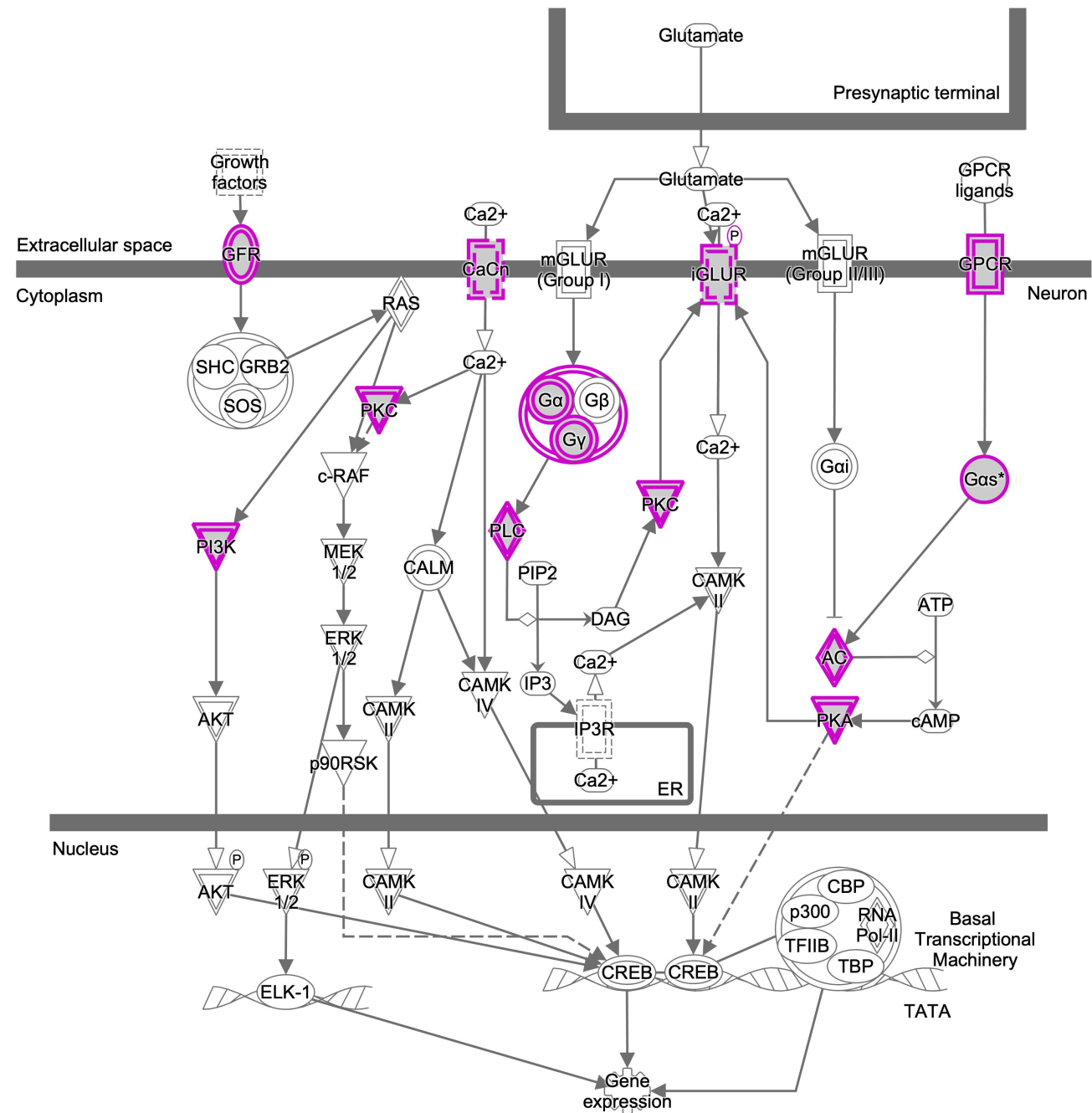

Figure S3B

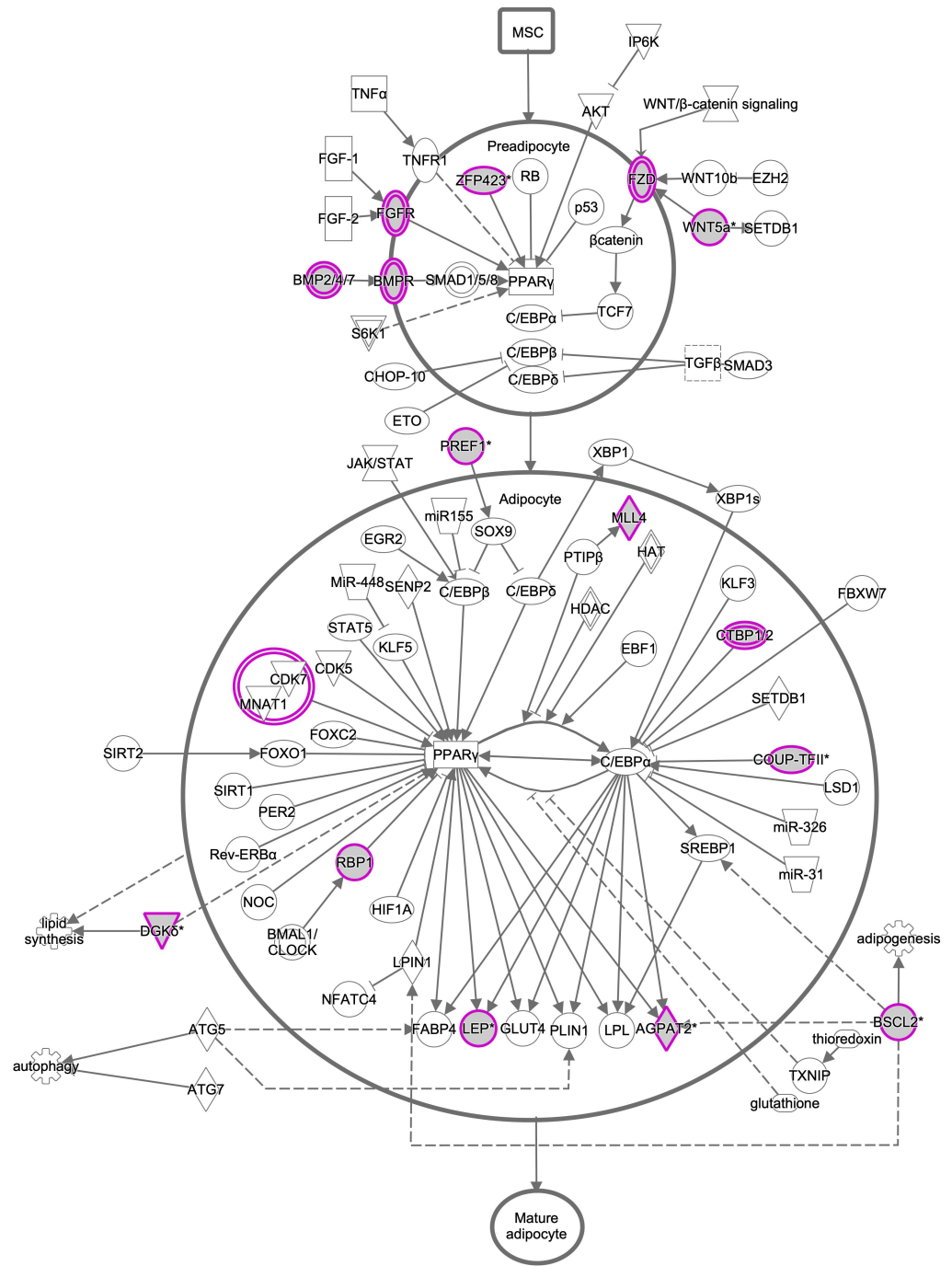

Figure S3C

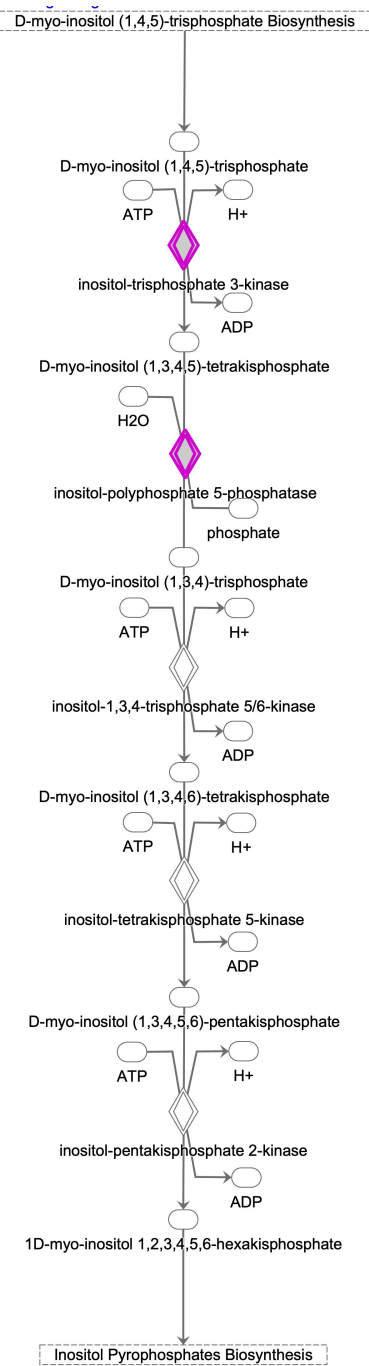

Supplement: Supplementary file 1 — Additional file 1. Figure S1: Enrichment of genes in (A) “RhoGDI Signaling” (p = 5.05 × 10^−6^), (B) “FXR/RXR activation” (p = 5.35 × 10^−6^) and (C) “Hepatic Cholestasis” (9.06 × 10^−5^). Figure S2: Enrichment of genes in (A) “molecular mechanisms of cancer” (p = 9.82 × 10^−9^), (B) “GNRH signaling” (p = 6.51 × 10^−6^) and (C) “TGF-Signaling” (p = 6.56 × 10^−6^). Figure S3: Enrichment of genes in (A) “Adipogenesis pathway” (p = 9.41 × 10^−6^), (B) “CREB Signaling in Neurons” (p = 4.25 × 10^−5^) and (C) “1D-myo-inositol Hexakisphosphate Biosynthesis II” (p = 4.41 × 10^−5^) [file 13148_2022_1266_MOESM1_ESM.pdf]
